# Study Protocol. Evaluating the life-course health impact of a city-wide system approach to improve air quality in Bradford, UK: A quasi-experimental study with implementation and process evaluation

**DOI:** 10.1186/s12940-022-00942-z

**Published:** 2022-12-05

**Authors:** Rosemary R. C. McEachan, Rukhsana Rashid, Gillian Santorelli, James Tate, Jamie Thorpe, James B. McQuaid, John Wright, Kate E. Pickett, Kirsty Pringle, Laura Bojke, Sally Jones, Shahid Islam, Simon Walker, Tiffany C. Yang, Maria Bryant

**Affiliations:** 1 Bradford Institute of Health Research, Bradford Teaching Hospitals NHS Foundation Trust, Bradford, BD9 6RJ England; 2 Institute for Transport Studies, University of Leeds, Leeds, LS2 9JT England; 3 St Stephen’s Church of England Primary School, Bradford, BD5 7HU England; 4 School of Earth and Environment, University of Leeds, Leeds, LS2 9JT England; 5 Department of Health Sciences, University of York, York, YO10 5DD UK; 6 Centre for Health Economics, University of York, York, YO10 5DD UK; 7 Bradford District Metropolitan Council, Bradford, BD1 1HX England

**Keywords:** Air Quality, Citizen science, Clean Air Zone, Interrupted time series analysis, Quasi-experimental evaluation, Health

## Abstract

**Background:**

Air quality is a major public health threat linked to poor birth outcomes, respiratory and cardiovascular disease, and premature mortality. Deprived groups and children are disproportionately affected. Bradford will implement a Clean Air Zone (CAZ) as part of the Bradford Clean Air Plan (B-CAP) in 2022 to reduce pollution, providing a natural experiment. The aim of the current study is to evaluate the impact of the B-CAP on health outcomes and air quality, inequalities and explore value for money. An embedded process and implementation evaluation will also explore barriers and facilitators to implementation, impact on attitudes and behaviours, and any adverse consequences.

**Methods:**

The study is split into 4 work packages (WP). WP1A: 20 interviews with decision makers, 20 interviews with key stakeholders; 10 public focus groups and documentary analysis of key reports will assess implementation barriers, acceptability and adverse or unanticipated consequences at 1 year post-implementation (defined as point at which charging CAZ goes ‘live’). WP1B: A population survey (*n* = 2000) will assess travel behaviour and attitudes at baseline and change at 1 year post-implementation). WP2: Routine air quality measurements will be supplemented with data from mobile pollution sensors in 12 schools collected by *N* = 240 pupil citizen scientists (4 within, 4 bordering and 4 distal to CAZ boundary). Pupils will carry sensors over four monitoring periods over a 12 month period (two pre, and two post-implementation). We will explore whether reductions in pollution vary by CAZ proximity. WP3A: We will conduct a quasi-experimental interrupted time series analysis using a longitudinal routine health dataset of > 530,000 Bradford residents comparing trends (3 years prior vs 3 years post) in respiratory health (assessed via emergency/GP attendances. WP3B: We will use the richly-characterised Born in Bradford cohort (13,500 children) to explore health inequalities in respiratory health using detailed socio-economic data. WP4: will entail a multi-sectoral health economic evaluation to determine value for money of the B-CAP.

**Discussion:**

This will be first comprehensive quasi-experimental evaluation of a city-wide policy intervention to improve air quality. The findings will be of value for other areas implementing this type of approach.

**Trial Registration:**

ISRCTN67530835 10.1186/ISRCTN67530835

**Supplementary Information:**

The online version contains supplementary material available at 10.1186/s12940-022-00942-z.

## Background

### The problem

Air pollution is one of the biggest contributors to mortality and morbidity globally, [1] surpassed only by health risk factors such as high blood pressure, tobacco use and poor diet [2]. Research has linked poor air quality with a range of health outcomes including poor birth outcomes [3]; cardiorespiratory disease [4]; lung [5] and non lung cancer [6]; and cognitive development and neurological disorders [7].

Research has estimated that 33% of childhood asthma cases are linked to poor air quality [8]. Emergency hospital attendances and mortality spike during periods of acute air pollution [9, 10]. In the UK, 64,000 deaths are attributable to outdoor air pollution each year, [11] with a greater burden of air quality related illness apparent in young people and the elderly [12]. The costs to the UK National Health Service (NHS) of treating air quality related illnesses between 2017 and 2025 is estimated to be £5.56 billion, [12] with the wider economic cost estimated to be £20 billion a year [13].

The UK is currently breaching legal limits of key pollutants such as nitrogen dioxide ([NO_2_], annual mean of 40 μg/m3) [14] and regularly exceeds World Health Organisation guidance (WHO) for other pollutants include particulate matter (PM) [15]. The burden of exposure is disproportionately borne by those of lower socio-economic status (SES), [16] and evidence indicates that health effects may be amplified by SES acting as a moderator between exposure and outcomes [17–19], thus increasing inequalities.

### Clean air zones (CAZ)

CAZs have been identified as potentially effective in reducing air pollution, [20, 21] and thus have the potential to improve health [22, 23]. CAZs are targeted at encouraging the replacement of vehicles with the newest engine and exhaust after-treatment systems, that the automotive industry now has to prove, are cleaner on-the-road (the Euro 6 vehicle emission standards and Real-Driving Emission legislation). In real urban driving conditions these vehicles can emit ten-fold fewer air pollutants (NO_2_, NOX, PM10 and ultra-fine particles) than their predecessors. In 2018, The UK Government issued ministerial directives to 28 local authorities to rapidly improve air quality to legal limits, including consideration of implementing a CAZ where older polluting vehicles are charged a daily fee to enter certain areas within a city or town [24].

Local authorities were asked to consider implementing one of four classes of charging for clean air zones which would see different tariffs for different types of vehicles which are deemed non-compliant with the requisite emission standards. A description of these classes can be found in Table 1. Class A, the least restrictive, would see charges for non-compliant buses, coaches and taxis. Class B would also restrict heavy goods vehicles and class C would see restrictions expanded to vans and minibuses. The most restrictive CAZ class (D) would see charge for all non-compliant vehicles including private vehicles. Local authorities were directed to conduct a range of modelling exercises to explore the likely improvement in air quality as a result of each different CAZ class and were advised to pick the most cost-effective of these options that would bring pollution to legal limits as quickly as possible. During this modelling period they were also asked to consider other activities (for example, park and ride facilities, electrification of traffic fleets) that could also be implemented to improve air quality.

**Table 1:** Charging clean air zone classes

Vehicle type	CAZ minimum emission standard	CAZ class			
		A	B	C	D
Buses and coaches	Euro VI	X	X	X	X
Taxis and private hire vehicles	Euro 6 (diesel) and Euro 4 (petrol)	X	X	X	X
Heavy goods vehicles	Euro VI		X	X	X
Vans and minibuses	Euro 6 (diesel) and Euro 4 (petrol)			X	X
Cars	Euro 6 (diesel) and Euro 4 (petrol)				X
Motorcycles	Euro 3				X

Although UK national health bodies have recommended implementation of CAZs to improve health, it is recognised that research exploring their effectiveness is lacking [21]. Systematic reviews highlight a lack of rigorous evaluation of CAZs or other similar initiatives on health outcomes [22, 23]. There have been few evaluations of interventions such as CAZs on air quality or health outcomes. Their impact on relationships between acute pollution episodes and short-term health outcomes has not been explored and little is known about whether these interventions can generate lifetime health and health inequality impacts and cost-savings [23]. In the majority of studies, modelled reductions in air quality (e.g. from projected vehicle emissions and air pollution dispersion models) are linked with assumed improvements in health [22, 23, 25]. Results from these complex causal modelling chains are highly uncertain and do not account for important real-world factors (for example, from elevated emissions of air pollutants from modern diesel vehicles, as exposed in the “dieselgate scandal”). Other weaknesses include lack of statistical power, no prospective follow up with baseline health data and lack of controls [26].

A recently published Cochrane review [27] containing studies up until 2016 identified only five studies linking interventions to reduce emissions from vehicular sources with health outcomes. Findings were mixed, and all evidence was rated as having low certainty. Three found positive effects of emission reduction on health: Yorifuji [28] found a 5.9% reduction in cardiovascular mortality and a 10% reduction in respiratory mortality associated with mandatory standards for diesel vehicles in Tokyo up to 12 years post implementation (reduction in particulate matter PM2.5 of 3.4%). Also in Japan, Hasunuma [29] found a 17.4% reduction in respiratory symptoms in children (aged 3 and under) from implementing vehicular standards for emissions (compared to 3.5% for children in control sites, reduction in NO_2_ 22%). El-Zein [30] found an immediate reduction in respiratory hospitalisations for children under 14 associated with a ban on diesel vehicles in Beirut, Lebanon. Russell et al. [31] found that implementation of pollution-control policies in the 5-county Atlanta metropolitan area (USA), which resulted in a decrease of 24 μg/m3 in NO_2_, led to a reduction of 5.9% of respiratory disease emergency department visits. In the Guanzhou region of China, Zhang et al. [32] found restrictions on emissions for the Asian Games resulted in a reduction of NO_2_ levels by 8.7 μg/m3, which decreased cardiovascular hospital admissions by 19.3% and respiratory admissions by 14.9%.

A study examining the impact of the London Low Emission Zone (LEZ, implemented in 2008) on health outcomes found modest improvements in NO_2_ (~1μg/m3), but no impact on children’s lung capacity, probably due to the small improvements in air quality [33]. No comparator was used and data were only collected after LEZ implementation. More recently, Pestel and Wozny [34] explored the impact of 58 low emission zones implemented across Germany between 2006 and 2016. They found the zones promoted moderate improvements in air quality reducing mean levels of key pollutants (PM10 and NO_2_) by 5%. Hospitals that were located within LEZ zones had small, but significant reductions in circulatory (reduction of 1.1% relative to a mean of 14% of cases, corresponding to 236 less cases per hospital on average) and lower respiratory disease (0.16% reduction relative to a mean of 1%, 34 fewer cases on average per hospital). None the studies identified explored the cost-effectiveness of the interventions in relation to health, and the impact of these type of interventions on populations’ attitudes and behaviours, including unintended consequences were rarely reported.

### The Bradford Clean Air Plan

Bradford, a city of located in the North of England in the United Kingdom, has been identified by the UK government as having a number of locations where the average annual concentrations of NO_2_ exceed the statutory limit of 40 μg/m3. As a result, the local government in Bradford (Bradford Metropolitan District Council, referred to hereafter as Bradford council) has been mandated by central government to bring about compliance in the shortest possible time, including consideration of implementing a charging CAZ. In response to this ministerial directive, Bradford council has developed and secured funding for the Bradford Clean Air Plan (B-CAP) and started to implement some activities, including provision of grant funding for local businesses to upgrade taxis, buses, lorries, coaches and vans in late 2021. The full B-CAP, which includes a ‘class C’ charging CAZ (please see below for further details) is planned to go live in 2022. The plan was informed by Government guidance, extensive modelling, consultation with business (e.g. bus and taxi companies), communities, including bespoke work with ‘seldom heard’ and ‘underserved’ communities [35] and local councillors.

### Aims and objectives

We aim to assess the impact of the B-CAP on attitudinal, behavioural, air pollution and health outcomes, and establish its cost-effectiveness, using a multi-outcome, multi-sector approach. We also aim to explore the factors influencing any impact (or lack thereof) and explore unintended or unanticipated outcomes. The impact of the B-CAP on health inequalities amongst different socio-economic and ethnic groups will be assessed across all outcomes. Our findings will enable the modelling of potential health impacts of a CAZ approach within other cities in the UK and internationally.

Our research questions are as follows:

1. What are the key barriers and enablers to implementation of the B-CAP (including acceptability), and are there unintended consequences of the B-CAP for different stakeholder groups (e.g., increased health and economic inequalities)?
2. Does the B-CAP affect travel choice behaviour and attitudes amongst people who live or work in Bradford at 12 months post implementation?
3. Does the B-CAP reduce exposure to pollution amongst primary school age children up to 12 months post implementation?
4. What is the impact of the B-CAP 3 years post-implementation on:
  - respiratory health (primary outcome, as assessed by weekly counts of respiratory disease related emergency hospital or General Practice [GP] attendance) of children (aged < 18), adults (aged 18-64) and older adults (aged 65+)
  - cardiovascular health (as assessed by weekly counts of cardiovascular disease related emergency hospital/GP attendance) of adults and older adults;
  - birth outcomes such as low birth weight and preterm birth (assessed by monthly counts)
5. How does the B-CAP impact on health inequalities up to 3 years post implementation?
6. What is the value for money of the B-CAP 3 years post-implementation and longer term?

## Planned intervention- the Bradford Clean Air Plan (B-CAP)

The Bradford Clean Air Plan (B-CAP), which includes a class C charging CAZ was approved by the Government on 12th February 2020 who awarded £43.3 million to Bradford council to implement the plan [36]. We describe the intervention in more detail in Table 2, using the standardised Template for Intervention Description and Replication (TIDierR) checklist [37].

**Table 2:** Description of the B-CAP including CAZ using the TIDieR checklist

Brief name	Bradford Clean Air Plan: Breathe Better Bradford
Why	To reduce NO_2_ pollution levels to legal limits (40 μg/m^3^) as quickly as possible. A Class C CAZ will charge non-compliant buses, coaches, heavy goods vehicles, vans, minibuses, taxis and private hire vehicles charged a daily fee to enter the zone. Proposed to instigate desired behavioural responses towards a reduction in most polluting vehicles and an increase public transport use, and active travel. Rigorous modelling (informed by routine monitoring and traffic fleet data) has identified at least 16 core link roads which exceed legal pollution limits, requiring a reduction of 1-18 μg /m3; activities included within the B-CAP have been estimated to achieve up to an 18 μg /m3 reduction in NO_2_ (based on 2018 baseline data). However, it is hoped that the ambitious nature of the plan will mean air quality is improved beyond these limits by harnessing the power of the ‘system’ (e.g. transport, planning, and public health) to work together to further improve outcomes.
What: Materials	Network of 330 automatic number plate recognition cameras, 16 km of digital ducting in 6 new digital rings around the city. A public website contains information for the public and businesses: https://www.bradford.gov.uk/breathe-better-bradford/breathe-better-bradford/
What: Procedures	The Clean Air Zone is planned to go live in 2022. Daily charges for non-compliant vehicles will be £7 for taxis, £9 for light good vehicles and £50 for heavy goods vehicles. Prior to the go-live date, local businesses and taxi are able to access grants to contribute to the cost of upgrading or replacing their vehicles to CAZ standards with 25% of all grants prioritised for electric vehicles. Exemptions will be provided for local small/medium enterprises (SMEs), schools and charities. The CAZ to be supported by a range of other initiatives including: electric bus routes in key parts of the city with road space allocation to prioritise buses and reduce journey times; installation of alternative energy centres providing cost effective green refuelling/recharging facilities; travel planning with businesses to promote car sharing, active travel and public transport use amongst employees.
Who provides	The intervention is implemented by the Clean Air Plan delivery team within Bradford council. This includes an operations team, grants and business support, monitoring and evaluation, and ultra low emission vehicle programme.
How	Drivers can choose to pay the daily charge up to 7 days before and 7 days after entry. ANPR cameras at CAZ entry points will identify non-compliant vehicles by linking up with the Driver and Vehicle Licensing Agency database. Penalty charge notices will be sent to owners who do not pay within 7 days. Grants and exemptions will be applied for via the Breathe Better Bradford website.
Where	The Clean Air Zone boundary encompassing the city’s inner ring road, and a key corridor out to the North West of the city. It encompasses an area of 22.4 km2, primarily the most deprived inner city wards but including less deprived wards on the outskirts of the city. The boundary contains ~ 20% of the Bradford population.
When and how much	The Clean Air Zone is planned to be live until legal levels of pollution are reached
Tailoring	Clean Air Zone interventions are complex and tailored within each city that they are implemented within.
How well	A number of measures will be used to explore whether the B-CAP is implemented as intended. These will include: fleet composition; number of penalty notices issued; number of vehicles upgraded; number of grants applied for

**Fig. 1:**
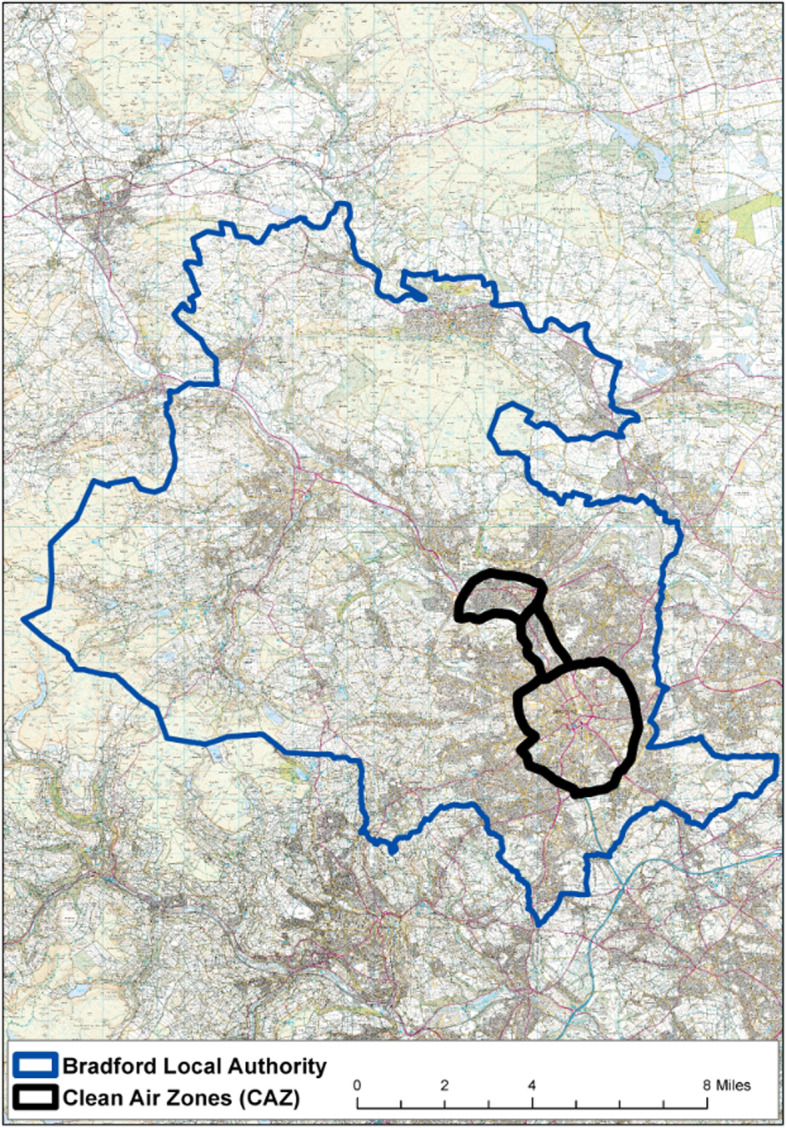
The Bradford Clean Air Zone boundary

A diagram of the CAZ boundary can be found in Fig. 1, and a logic model summarising the proposed intervention and its hypothesised impacts on outcomes can be found in supplemental file 1.

## Methods

### Design

**Fig. 2:**
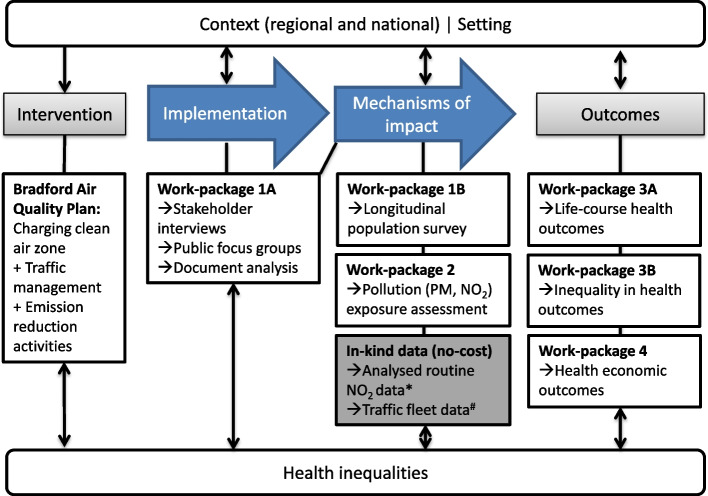
Outline of conceptual framework (adapted from MRC Guidance for Process Evaluation of Complex interventions) NB *: Analysed data provided via DEFRA national evaluation [39]; ^#^ Anonymised vehicle Fleet composition Data are provided by Department for Transport who process the data from the Bradford MB Council Automatic Number Plate Recognition system

Our evaluation is based on the MRC guidance for the evaluation and process evaluation of complex interventions [38] and is structured around 4 core work-packages (WP) to assess implementation, mechanisms of impact, health and economic outcomes (see Fig. 2). We will explore how context interacts with and influences intervention delivery and outcomes, and the impact of the intervention on health inequalities. Our quasi-experimental approach will capitalise on a natural experiment within the city (implementation of the B-CAP) and exploit a unique research infrastructure including the Connected Bradford (cBradford) data set of > 530,000 Bradford residents, [40] detailed surveys and longitudinal health assessments of > 12,500 families participating in the BiB birth cohort study [41, 42]..

### Setting/ context

Our study is located in Bradford, an urban, multicultural city in the North of England, UK. Bradford is the 6th largest metropolitan district in the UK with a population of > 530,000. It has a multi-ethnic population, with 67% identifying as White British and 20% as Pakistani [43] and with increasing numbers of families arriving from Eastern Europe [44]. It is a deprived city, with 40% of Bradford residents living in areas that rank in the most deprived quintile (20%) of local areas in England [45]. It has high levels of ill health, e.g., higher than average mortality from cardiovascular disease under 75 years (102.2 per 100,000), low birth weight babies (3.6%), [46] and 22% incidence of wheezing disorders amongst children [47].

### Study population

For our primary analysis (WP3A), we plan to take a whole city/district, life-course approach. Using our connected data set of linked routine health data from over 500,000 Bradford residents we will explore outcomes across the population, before stratifying into three groups (children: at birth and 0-17 years; adults: 18-64 years; and older adults (65 years+). Key health outcomes will be extracted from this dataset to provide a comprehensive analysis of any effect of the B-CAP on population health outcomes.

In addition to this whole city evaluation we will harness the rich data from the BiB cohort of 12,500 Bradford families and 13,500 children for whom we have detailed longitudinal health and wellbeing assessments (including routine data) and rich socio-economic and ethnicity information. This cohort will allow us to provide more detailed insights into the effect of the B-CAP on health inequalities, and to interrogate our proposed logic model. BiB is a representative research-active cohort of families with children born in the city between 2007 and 2011 (~ 60% of the eligible population at time of recruitment). Approximately 85% of the cohort is still resident within Bradford. Fifty percent of mothers in the cohort are of South Asian origin, which was broadly representative of births in the city during the period of recruitment [41]. All of the parents in the cohort are of working age. Routine linked health data is available for 99% of parents and children (which also allows geocoded address information to be captured monthly from primary care records), and education data is available for 85% of children. In addition to these large datasets, other elements of the evaluation will focus on bespoke data collection with key groups including decision makers and stakeholders who have been involved in implementation of the B-CAP plans; and with members of the public, schools and families living in and around the CAZ boundaries.

### Work package (WP) 1- Implementation evaluation

We will employ a mixed methods approach to conduct a process evaluation, [38] which draws on recent advances in conceptualising adaptations to intervention processes, [48] and explores relationships between context, implementation and setting [49]. This will help us interpret why the B-CAP has (or has not) affected outcomes and inequalities and allow us to understand unanticipated or adverse outcomes.

#### WP1A: Process and implementation evaluation

We will construct a ‘systems’ map to guide our data collection process. This will involve working with members of the Bradford Air Quality Programme Board to map out key partners, organisations and systems which may impact on, or be affected by implementation of the B-CAP. In WP1A, we will gather qualitative data to inform an implementation and fidelity evaluation to build a picture of the degree to which the intervention was delivered as intended, acceptability and unanticipated or adverse events. In WP1B, we will gather quantitative data via a longitudinal population survey to explore mechanisms of change, specifically whether the intervention has the expected changes in behaviours and attitudes up to 1 year post implementation. We will supplement this bespoke data collection with available information collected via the Bradford council ANPR survey assessing vehicle fleets (proportion of compliant vehicles) travelling within Bradford, and the CAZ boundary. All data sources will allow us to test the assumptions and mechanisms in our logic model and thus, our implementation pathways.

In WP1A core components of data collection will include i) documentary evidence, ii) semi-structured interviews with stakeholders, iii) focus groups with members of the public, and iv) semi-structured interviews with key people involved in the implementation of the B-CAP. We will collate relevant documentary evidence which will allow us to record in detail how the intervention was implemented (for example, air quality board reports and minutes, council scrutiny reports and minutes), and the relevant regional and national policy context (for example, policy briefings, government reports). Semi-structured qualitative interviews will be conducted before the CAZ is operational (for implementers) and at 12 month follow-up with key stakeholders (from businesses, including small to medium enterprises [SME], travel sector, local authority, and voluntary sector) to explore barriers and facilitators to implementation, and reasons for intervention adaption. We will use purposive sampling methods to recruit participants from the local authority (primarily the Bradford Air Quality Programme Board, responsible for intervention development and implementation), local business (Bradford Chamber of Commerce), transport (bus and taxi) operators and local action groups. We will recruit to data saturation but anticipate conducting ~ 20 interviews pre implementation and ~ 20 interviews post implementation. Workshops with stakeholders who have strategic insight of implementation and system issues will be conducted at 2 and 3 years post-implementation to identify key barriers and enablers.

Public engagement, reach and responsiveness will be assessed via focus groups to explore the degree to which intervention implementation has impacted potential beneficiaries and to explore the potential mediating process through which air quality interventions may impact on health outcomes. Participants will include members of the public, including school staff/teachers, parents/children, commuters within and outside the CAZ boundary. We will work with local voluntary sector organisations and schools to identify and recruit a diverse (age, ethnicity, SES) representative range of participants. A discussion guide will be developed covering issues related to acceptance, attitude, impact, engagement, reach and responsiveness. Informed consent will be taken prior to interviews / focus groups which will be audio-recorded and then transcribed. Expenses and refreshments for participants will be provided.

#### WP1B: Population survey

We will use the BiB cohort as a core sampling frame for our longitudinal survey, in addition to surveying members of the general public. Benefits of using the BiB cohort include the detailed longitudinal information available on health and wellbeing for parents and children, existing consent to contact participants, and validated address and email contact details. The survey will measure changes in travel choice behaviour following B-CAP implementation, and attitudes and participants’ views of the intended and unintended impacts of the B-CAP components on health and travel behaviour (see logic model in Supplemental File 1). Given that our research has coincided with the COVID-19 pandemic, we have adapted our planned survey to also include key questions in relation to people’s experiences of the COVID-19 pandemic and subsequent local and national lockdown restrictions. Specifically, items related to health and wellbeing, financial circumstances, changes to transport and travel behaviours and family priorities for healthy and happy children have been added. A copy of the questionnaire can be found in Supplemental File 2.

The population survey will be administered immediately pre, and up to 12-month post implementation of the charging CAZ element of the B-CAP. All BiB families still living within Bradford district will be eligible (~ 10,000, 80% of total cohort). We will offer the survey in a variety of formats including paper based, online, and face to face with multilingual researchers facilitating completion in Urdu, and Mirpuri. The survey will be promoted to eligible families using a variety of well-established recruitment methods, including direct communication (letters in school book bags, post) and wider promotion (social media, website, and newsletters). When the study was originally planned, pre-pandemic, we had aimed to recruit 4000 families (40% response rate). However the COVID-19 pandemic severely curtailed our research activities, and like many other studies we found that participant’s burden increased, impacting on their ability to take part in research. Based on this we revised our estimate to aim for 2000 responses at both time points, and plan to make the survey available to the wider population of Bradford. We will advertise through a variety of online social media channels including and via council communication channels (e.g. email lists, twitter). We will attend community venues and events with computer tablets where participants are able to access the online questionnaire, and paper version of the questionnaire.

#### Analysis methods

In WP1A we will explore the implementation and fidelity of the intervention and determine the extent to which any adaptations affect the functioning principles (described in the logic model) of interventions or their components. For example, assessing categories of ‘what’ (e.g. reduction in numbers of non-compliant vehicles); ‘how’ (e.g. provision of addition public transport infrastructure; incentives schemes to replace vehicles); ‘to whom’ (families/children; business); ‘by whom’ (e.g. council, contractors, public representatives), and whether any (dis) benefits of the intervention are spread equally amongst different socio-economic groups. Qualitative data from the stakeholder interviews, implementation interviews and public focus groups will be analysed separately using thematic analysis [50]. Stakeholder interviews will focus on identifying barriers and facilitators to intervention implementation; the COM-B model [51] will be used as a conceptual model to categorise identified barriers and facilitators. Process implementation data (e.g. documentation forms, meeting minutes) will be reviewed by the project management team, and intervention components categorised into adaptation levels (implemented, not implemented, modified). These will be tabulated, scored and presented descriptively. Documents collected to inform contextual influences will also be analysed using thematic analysis. All forms of implementation and adaptation evaluation data will be used to provide a picture of which (and how) adaptations impacted on which outcomes in relation to the intervention descriptors. Data from our population survey (WP1B) will support an understanding of the mechanisms of any change and will compare travel/air quality attitudes and behaviour pre and post implementation using McNemars test, paired t-test and Wilcoxon signed rank sum test, stratified by participant characteristics such as ethnicity and socio-economic status. We will be able to examine changes for respondents living within and outside CAZ boundaries to explore whether any changes in behaviour or attitudes differ according to proximity to the CAZ boundary. We will combine insights from WP1A and WP1B with relevant data from Bradford Council (e.g. the ANPR survey) to critically review the proposed logic model (see supplemental file); including developing a ‘dark’ logic model [52] outlining key barriers to implementation and threats to the intervention logic to explain any observed unanticipated or adverse events. Results of the implementation evaluation will be considered alongside the evaluation of effectiveness for health outcomes to provide context to help explain findings (including use of implementation data as an effect modifier). This will be done during discussion with the immediate team, wider stakeholders and our community public involvement groups.

### Work package 2: Air quality

We plan explore mechanisms of impact of the B-CAP on air quality using routinely collected air quality monitoring data, supplemented by citizen science collected air quality data around schools. We are able to add significant added value to our analyses by leveraging the extensive routine monitoring data, analysis protocols and outputs which are being undertaken as part of a national evaluation funded by DEFRA [39]. This will examine the 28 local authority areas (including Bradford) who are developing air quality plans and associated control sites. Using routinely collected air quality and traffic data the analysis will explore the impact of the clean air plans on pollution over and above underlying trends in air quality, traffic demand, transport use and the evolution of the vehicle fleet. Key outcomes will assess whether the B-CAP impacts on air quality. They will include routinely monitored air pollution (assessed via DEFRA national evaluation at 1 year and 3 years follow up) and mobile-sensed personal exposure to air pollution (assessed up to 6 months follow up).

#### Sampling and data collection methods

##### Routine air quality data

Council led routinely-collected hourly data of NO_2_ from seven continuous real-time monitoring stations (five of which are within the zone) will be extracted over the study period, supplemented by 400 NO_2_ Palmes diffusion tubes. Three of the continuous monitoring stations also measure PM_10_ and PM_2.5._ Data will be collated for 3 years prior, and 3 years post implementation.

##### Citizen science monitoring

Children are particularly vulnerable to the harmful effects of pollution, [53] and much of their exposure is experienced on their daily commute to and from school [54]. Children can be powerful agents for change, and as part of our planned evaluation we were keen to harness the enthusiasm of communities and young people by including them as ‘citizen scientists’ who could contribute to our research activities. Together with our public contributors we developed an air quality monitoring protocol which would involve primary school age pupils (aged 9-11) carrying mobile sensors on their journey to and from school. The aim is that these activities can be used to educate and inspire young people in issues regarding air quality and research.

We plan to recruit 12 schools to take part in citizen science air quality measurements which will involve installation of static sensors and diffusion tubes in key school locations recording PM and NO_2_, and ‘intensive observation periods’ where mobile sensors are carried by children during their school week and commute to school. Four schools will be located within the CAZ boundary, 4 schools will be located just outside the CAZ boundary (to explore any potential ‘displacement’ of pollution caused by drivers taking alternative routes to avoid the CAZ), and 4 schools will be distal (> 2 km) from the CAZ boundary.

We had planned that monitoring take place over a 24-month period (encompassing the year prior, and the year post CAZ implementation), however, lockdowns and school closures associated with the pandemic has meant this monitoring period has been reduced. For the intensive observation period we are now planning to measure children’s exposure at two time points pre-implementation (Autumn 2021, Spring 2022) and two time points post implementation (Summer 2022, Autumn 2022).

Within each school we will recruit 20 citizen scientists (total of *N* = 240) to measure pollution on their commute to and from school during parallel data collection periods across the 12 schools. Each pupil will carry the sensor for up to 5 days during each observation period, with data collected over 4 week observational periods within each school (a total of 20 days per pupil over 12 months). Atmotube Pro sensors (https://atmotube.com/products/atmotube-pro) which measure PM, volatile organic compounds (VOC), temperature and humidity, will be linked to a research smartphone which will also collect GPS tracks for their walk to and from school; these devices will be carried in a small waist/belt-bag. Children will be encouraged to engage in normal activity throughout the week. They will complete a short diary to record how they travelled to school, and locations visited each day. Detailed instructions and training will be given to pupils and parents.

Static sensors have been developed which use a Raspberry Pi Zero connected to two PM sensors: a Sensiron SPS30 PM sensor (which measures PM1, PM2.5 and PM10) and an Alphasense R2 optical particle counter which also gives more detailed particulate matter size information. Ambient temperature and relative humidity are also measured. Atmospheric data is logged every 10 seconds. Each school has the option to receive 3 static sensors; one outdoor and two indoor (one in a classroom and the other in a central location normally the dinner hall. All schools also have the option to receive a commercially available Purpleair PM2.5 sensors which displays data on a simple user friendly interface (https://map.purpleair.com/1/mPM25/a10/p2592000/cC0#10.89/53.7837/-1.7868) which is an effective tool for teaching and engagement. Triplicate NO_2_ tubes, which are changed monthly are installed at key outdoor locations for each school. A web dashboard to allow easy visualisation of pollution data collected via static and mobile sensors will be developed.

#### Analysis methods

Routinely collected air quality data will be analysed by the DEFRA national evaluation team in parallel to the current evaluation and will allow an analysis of trends in air quality across the district. The approach estimates the time-varying background levels of air quality from the Automatic Urban and Rural Network (AURN) of continuous monitoring stations for any given time point, and subtracts these from locally measured air quality data at roadside locations so that the contribution and trends associated with local traffic and the implementation of the B-CAP can be identified. As air pollution levels vary with fluctuations in emissions and dispersing air-flows, the underlying changes can only be established by ‘de-weathering’ and ‘de-seasoning’ local data [55]. Any abrupt or gradual changes in these trends around the intervention date of the B-CAP will then be estimated using change point detection methods [56].

The DEFRA evaluation methods for processing data will be adapted to deal with the additional data collected across our 12 schools within our intensive observation period (mobile sensing with *n* = 240 citizen scientists) and extended observation periods (continuous monitoring from static sensors).

The personal exposure of school citizen scientists (*n* = 240) before and after the introduction of the B-CAP will be mapped onto a 500 m × 500 m grid centred on each school. Any difference in levels, over the under-lying trends (as determined by the DEFRA evaluation project), will be attributed as impacts of the B-CAP. If the intervention is successful, it is expected that any differences would be greatest for the schools located within the CAZ boundary; however, as the CAZ may influence overall traffic volume and traffic flow, the benefits may extend beyond the CAZ boundary. Our study design allows us to explore any dose-response relationships with schools located on CAZ boundaries and distal to the CAZ boundary. We will explore differences using a 2 (pre/post) × 3 (location: inside, bordering or distal to CAZ) design using the Friedman test. Data from personal sensors will also be used to create an ‘exposure index’ along key transit routes to and from schools, allowing us to explore impact of the B-CAP on exposure during the school commute.

For the extended observational period (static monitoring) we will generate an understanding of pollutant distribution in the area, we will use data interpolation between the sites alongside the annual pollutant profiles close to the project schools.

### Work package 3: Health impacts

We will assess the extent to which implementation of the B-CAP impacts on health outcomes, using a quasi-experimental interrupted time series design with our Connected Bradford (cBradford) and BiB data sets. We will compare trends of health outcomes collected for 3 years pre, and 3 years post implementation (minimum 6 years data). Using the cBradford dataset, in WP3A we will explore changes in a range of health outcomes across the life-course up to 3 years post implementation using a segmented regression approach. Drawing on the PROGRESS framework for health equity [57], in WP3B we will investigate in detail the impact of the B-CAP on health inequalities, considering a wide range of characteristics (for example, household-level SES and ethnicity), focusing on respiratory health outcomes for 13,500 children in our BiB data set.

#### Power calculations

In order to explore power of the ITS analysis to detect changes in health outcomes we have consulted a range of epidemiological studies and systematic reviews, along with identification of relevant intervention studies for effect sizes. Our own research has found that a 10μg/m3 increase in NO_2_ is associated with a 9% increase in the odds of low birthweight, while a 5μg/m3 increase in PM2.5 is associated with an 18% increase in odds of low birth weight [58]. For other health outcomes, systematic reviews have shown a 10μg/m3 increase in NO_2_ to be associated with increases in respiratory disease hospital admissions ranging between 0.57-3.5%, and a 0.66% increase in admissions for cardiovascular admissions, with stronger effects among children and the elderly [59–63].

Recent intervention studies (reviewed earlier) offer no consistent results. Based on the evidence reviewed and working on the assumption the B-CAP will reduce NO_2_ by between 12-18μg/m3 we have estimated a 5% reduction in health outcomes (emergency / GP attendances and incidence related to both respiratory and cardiovascular outcomes); and a 2% reduction in adverse birth outcomes. A 5% reduction in emergency attendances in relation to respiratory and / or cardiovascular illness would likely have substantial cost-savings for the NHS [64].

We have reviewed methods to inform power calculations; there are no accepted guidelines for calculating power in ITS designs [65, 66]. Power is dependent on a variety of factors including the length of the time series (with some suggesting 100 observation points required for correct model identification), [67] the balance of time points before and after the intervention, the expected effect size, the extent of autocorrelation between the data points, and sample size per time point. Power increases in a balanced ITS design; in WP3 our proposed study would increase the power by ensuring roughly equal number of data points for the 3 years pre- and 3 years post-implementation. We illustrate the potential power of our analyses for our primary outcome, respiratory health (measured as emergency/GP admissions): in pilot analysis of a subsample of our connected data set (*N* = 316,585, assessed between Jan 2016-Dec 2018) we observed an average of 380 respiratory attendances per week suggesting adequate counts per observation period. Using weekly counts will give us a total of 312 observation points over a 6 year period. Guidance from simulations of Winquist et al. [66] suggests we will have > 90% power to detect a 2% reduction in admissions. For our secondary outcomes (cardiovascular/birth) we will assess counts either weekly or monthly depending on prevalence. Using monthly events a balanced design would give us 72 evenly distributed observations points. We will explore increasing the baseline intervention trend period by 28 months (e.g. 5 years pre-implementation) to ensure we can maintain 100 observation points (which is acceptable if there are no other major changes in pre-intervention trends in that time period) [65].

In WP3B we will focus only on respiratory attendances within the BiB cohort using similar methods. For these analyses we will have fewer events per observation period and so will perform analyses using either weekly or monthly counts.

#### Health outcomes measures

We have selected a range of health outcomes to explore, informed by epidemiological evidence. Our primary health outcome will be changes in respiratory health outcomes assessed by Accident and Emergency (A&E) and GP attendances overall and stratified by age group: children (0 – 17 years), adults (18 – 64 years), and older adults (65+). Our secondary analyses will examine cardiovascular outcomes in adults (stratified by age groups defined above), and birth outcomes (pre-term birth and low birth weight), see Table 3.

**Table 3:** Description of health outcomes

Health Outcomes	CTV3 and ICD-10 codes	Length of follow up	Life-course stage
**PRIMARY**: Respiratory health: A&E / GP attendances, assessed weekly	CTV3 codes: XaKyZ, Xa9Bt, X00n8, X00n9, H27.., H270., XM0rz, XaIBK, H2700, X100E, Xa0lY, XE0YG, XaDsa, X100H, Xa7nL, H200., X100J, XM0rv, Xa7nU, Xa7nT, Xa7nP, XE0YH, XaJEl, Xa7nL, Xa7nN, Xa7nM, X100M, XE0Xt, XE0Xs, XE0Xr, X100B, X1007, XSDOK, H061., X100C, H0615, XaYYt, XaDtP, H30z., H31.., H310., XaDtg, Xa87h, H32.., H3..., H3z.., XaEIV, XaEIY, XaEIW, XaN4a, H33.., X101x, XE0YX, 663 V1, H332., 663 V2, 663e., 663P., 663 N2, XaXZx, XE0YW, 663 N0, 663 N., 663 N1 ICD-10 codes: Respiratory infection (J05.0, J10-J16, J18, J20, J21); bronchitis (J40 – J42); asthma (J45); COPD (J43-J34)	1 year (interim) and 3 years post implementation	Children Adults Older adults
**SECONDARY**: Cardiovascular health: A&E / GP attendances, assessed weekly/monthly	CTV3 codes: XE2uV, G3z.., G33.., G33z., Ua1eH, X200E, XE0Uh, G35.., G30y., G305., G301z, G304., Xa0YL, G308., XE0WC, G3110, G312., G70.., XM0rN, XE2uV, XE2aA, G341., XM1Qk, G3410, G3412, G343., X200D, XE0WG, G34.., G34z., X202Z, G561z, XE2QF, X202a, G560., G5610, X202d, G5620, XaRCL, Xa0lU, G565., G564., X77Ab, G5654, X202Y, XE0We, G56zz, XE0V5, X202n, XaC2L, X2025, G570., XE0V4, G572z, G5730, G573., G5740, G574., G58.., XE0V9, X202l, XE2QG, XE0V8, Xa1uW, G60z., XE0VF, G61z., XA0BG, Xa0kZ, XE0VJ, X00D1, XaEGq ICD-10 codes: Angina/MI (I20-I22, I24, I25); dysrhythmia/conduction disturbance (I44-I49); heart failure (I50); stroke (haemorrhage or infarction; I60-I64))	1 year (interim) and 3 years	Adults Older adults
**SECONDARY**: Birth outcomes, assessed monthly	ICD-10 codes: Pre-term birth (< 37 weeks gestation: O60.1, 060.3, P07.2, P07.3) and low birth weight (< 2.5 kg: P07.0, P07.1)	3 years	Children

We will explore health inequalities in detail for respiratory health amongst children using the BiB data set.

#### Sampling and data collection methods

Data extraction algorithms for health outcomes will be compiled using WHO International Classification of Disease (ICD) 10 and CTV3 Read codes and applied to our cBradford and BiB data sets. Relevant CTV3 codes will be identified by mapping them to ICD-10 codes using the NHS Digital Technology Reference data Update Distribution. We will extract a range of other characteristics recorded routinely in health records including gender, age, and ethnicity to characterise outcomes along social gradients. Data will be linked to Lower Super Output area to calculate the Index of Multiple Deprivation (IMD), a measure of relative deprivation. Within the BiB cohort, in addition to linked routine data, we have detailed ongoing assessments of health, wellbeing, cognitive development and socio-economic circumstances currently underway amongst children aged 7-11, [42] along with monthly geocoded address data. This wave of data collection was completed in March 2020 (prior to implementation). This detailed baseline information will allow detailed subgroup analysis to be performed in WP3B to explore the impact of the B-CAP on health inequalities using dimensions such as SES (education, IMD, financial security and employment status) and ethnicity.

#### Analysis methods

For WP3A, demographic information from cBradford (age, sex, ethnicity, IMD and geographic location (ward level of those inside and outside the CAZ)) will be summarised using frequency (%) or mean (SD). For WP3B, information (age, sex, ethnicity, IMD, measures of SES) from the BiB cohort will similarly be summarised. An interrupted time series (ITS) design using segmented regression analysis will be used to estimate levels and trends during specific points in time (segments), where the values of the time-series may change from the previous pattern because changes in air pollution trends as a result of the B-CAP intervention. In segmented regression, terms are included in the model to indicate, for example, the baseline level of the outcome, the implementation of the intervention, and time after the intervention. These effectively fit separate regressions for “segments” so that it is possible to estimate changes in level and trend. The assumption underlying our analyses is that we would expect a slope change in our outcome measure as we expect the implementation of the B-CAP and its effects are gradual.

We will examine time-series line plots of the health outcomes to aid identification of underlying trends and seasonal patterns of the data. As the outcomes are counts, the Poisson link function will be used in the regression model. Models will be adjusted for age, sex, ethnicity, and measures of SES such as IMD. The coefficients from this regression can be used to estimate the intervention effect (the absolute difference between the predicted outcome based on the B-CAP and its counterfactual value, i.e. the expected values for level and trend if the intervention had not occurred. We will account for the disruption caused as a result of the COVID-19 pandemic, and the subsequent changes in behaviour including alterations to travel behaviour and A&E and GP attendances, through the selection of change points used in the segmented regressions. In England, the first lockdown was implemented on the 23rd of March 2020 and the third ended on the 31st July 2021. We will therefore consider four segments for our analysis: (1) the period prior to the first national lockdown from when clinical data of sufficient quality was available (October 2017 – March 2020); (2) the period spanning the lockdowns (March 2020 – August 2021); (3) the period encompassing the preparation phase for the CAZ (August 2021 - CAZ switch on, this includes other activities within the B-CAP including provision of grants for vehicle upgrades); (4) the period post ‘switch on’ of the charging CAZ). We will conduct an interim analysis at 12 months post-implementation and re-examine analyses 3 years post-implementation.

Given the seasonal variation in respiratory outcomes and pollution, data will be adjusted for seasonality. We will also assess the models for residual autocorrelation and apply ARIMA methods as necessary. We hypothesize that the B-CAP will lead to a larger decrease in outcomes for those living in more deprived areas and by ethnicity and will examine these through interaction terms and stratified analyses. We will consider a series of sensitivity analyses by varying model assumptions. We expect air pollutants to decrease the most within the CAZ boundary compared to without and will examine the impact of spatial variation on outcomes. We will test our assumption that the effects of the implementation of the CAZ are gradual, by altering the length of the third segment based on dates of when implementation activities were initiated. We will also assess lagged effects between exposures to air pollution and health outcomes.

ITS is a strong evaluation design when randomization is not possible as post-intervention trends can be compared to trends prior to the interruption of the intervention. We will strengthen this design through the inclusion of a non-equivalent control (NEC) outcome (superficial head injuries) which will not be affected by the intervention (CAZ) to control for possible concurrent events. Previous studies utilizing a NEC have used finger wounds [31]); we chose superficial head injuries given the higher number of cases per month compared to other potential injuries. A change in the outcome of interest following implementation of the CAZ with no change in the NEC would increase our confidence that the changes observed were a result of the intervention.

For WP3B, we will conduct detailed analyses on health inequalities using data from the BiB cohort, which has individual, in addition to area level, information on socioeconomic status. We will replicate analysis on respiratory outcomes looking at both combined respiratory morbidities and separately at asthma/wheeze, exploring multi-dimensional measures of socio-economic status, [68] ethnicity, deprivation, employment, religion, education and social capital using the PROGRESS framework as a guide to selecting relevant indicators [57].

### Workpackage 4: Cost effectiveness analysis

Following guidance on appropriate methods for economic evaluation alongside natural experiments, [69] and our recent work, [70] we will perform a multi-sectoral cost-effectiveness analysis to determine value for money of the B-CAP considering a range of costs across sectors, and quantifying outcomes in terms of quality adjusted life years (QALY). Key economic outcomes will include healthcare resource use and costs, private economy and LA costs, quality adjusted life years, distributional effects (all at 3 years follow up). The comparator in our evaluation will be pre B-CAP implementation outcomes and costs. Our evaluation will explicitly consider the costs and outcomes for a range of decision makers (for example, health care, private economy and local authorities) and sectors (e.g. business, health), along with different socioeconomic groups to capture distributional effects (e.g. burden of paying charge) which may impact on inequalities. We will consider the cost of delivering the interventions in the B-CAP, and any cost-offsets, for example due to reduced GP consultations and hospital admissions.

We will consider the relevant costs, opportunity costs and benefits for each decision maker/sector involved. Potential sources of double counting will be explored and adjusted for where possible. We will then summarise the impacts on each sector, for example, the impact on health and health care costs from an NHS perspective. An aggregate approach across all sectors will also be considered with costs and benefits brought together to estimate the societal benefit of CAZ, which can be expressed as Net Benefit (benefits net of any opportunity costs). Different time horizons will be considered for the analysis to account for budget cycles, differences by sector, i.e. cost may have to be justified over a short time period for some sectors, including local authorities. .

The overall cost of the B-CAP will be estimated, and we will show how much of this cost falls on local authorities, and how much falls in the wider economy where they cascade to individuals and private companies (e.g. cost of charge for non-compliant vehicle). The costs of the B-CAP to the local authority and the wider economy, will be assumed to continue for as long as the intervention is applied. Wider societal benefits will be considered by exploring any impacts on productivity and individuals’ private consumption related to health status, i.e. a health event incurs a productivity loss and associated cost to the economy in terms of lost wages and may reduce an individual’s private expenditure [71].

The analysis of CBradford data will also allow us to consider the change in health across different socioeconomic groups (defined by the Index of Multiple Deprivation of the population of the area in which people live at Lower Layer Super Output Area).

Distributional cost-effectiveness analysis [72] will be used to evaluate the impact the B-CAP has on the socio-economic distribution of health, reflecting both the direct impact of the B-CAP on the health of different socioeconomic groups and the impact on heath in terms of forgone health from opportunity costs resulting from any costs associated with the B-CAP. The value for money from the B-CAP can be evaluated considering both goals of improving total population health (net benefit in terms of QALYs for the population of Bradford) and reducing inequalities (e.g. reducing the gap in health outcomes between the most versus the least deprived groups in Bradford, or between ethnic groups).

The within study analysis will utilise data from WP3 with a 3 year follow up. In addition, the economic evaluation will extrapolate any changes in short term outcomes to longer term costs and consequences using a decision analytic model. For changes in short term health outcomes, for example cardiovascular, respiratory disease and incidence of pre-term births, these health states will be extrapolated over the longer (life) time, to determine the impact of these conditions on quality adjusted life years (QALY) and costs. This lifetime modelling work will also reflect costs to all sectors impacted by the CAZ and the impact on inequalities. The model will also reflect the sampling uncertainty in the evidence available from WP3, the costs of the CAZ from WP1 and any external evidence used to extrapolate health states. We will use probabilistic cost effectiveness analysis to establish the amount of uncertainty in our value for money assessments.

### Narrative synthesis of findings

Evaluation of complex interventions such as the B-CAP requires a comprehensive strategy. Our conceptual model (Fig. 2) recognises the links and interactions between all work-packages. For example, in order to understand the impact of the B-CAP on health inequalities we will combine insights from qualitative research (WP1A), survey (WP1B), air quality assessment (WP2), health outcomes (WP3) and our health economic analysis (WP4). In order to explore bidirectional influences of context and setting we will consider our findings in light of policy changes, new information (for example, national trends in air quality, vehicle ownership) and information from the DEFRA national evaluation over the duration of the study. We will synthesise findings from each work-package to develop a broader and comprehensive analysis which will help us to develop recommendations to inform policy.

### Follow-up

We have included a range of outcome measures that are likely to reflect shorter (A&E/GP attendances related to respiratory and cardiovascular disease), and longer term effects (birth outcomes). The B-CAP is mandated to improve air quality to compliant levels in ‘*as quick a time as possible’*. Based on modelling, the likely impact of measures included within the CAZ, compliance is due to be achieved 1 year post implementation, with gradual ongoing improvements over time. However, research has shown there is no ‘safe’ level of pollution for health, thus any improvements in air quality are likely to have an impact on health, regardless of whether they are above, or below legal limits [58]. Evidence has shown an immediate temporal relationship between increases in pollution and hospital attendances [60] thus we would expect to see an immediate reduction in emergency and GP attendances related to respiratory (adults and children) and cardiovascular (adults only) events provided the B-CAP is successfully implemented. Our other outcomes (birth outcomes) are likely to show an effect in the longer term. We have chosen 3 years as our primary follow-up period to measure effectiveness (with an interim reporting period at 1 year) period in order to provide timely evidence for decision makers, including other local authorities on the health impacts of CAZ approaches to improving air quality. As our primary analysis relies on routine data we have the unique opportunity to revisit our study populations beyond the lifetime of the planned research to provide even longer term follow-ups of the health outcomes outlined in Table 3. We have chosen a 1 year follow up for our process and evaluation outcomes to ensure timely capture of challenges related to implementation, and identification of the proximal effects by which the intervention might have an impact on outcomes.

### Unanticipated outcomes

During our stakeholder and public consultations we have identified concerns about potential adverse outcomes [35]. For example, there is a perceived risk of displacement of pollution to the borders of the CAZ as non-compliant vehicle operators choose to take different routes to avoid a charge. There is also the potential for increasing inequalities by economically disadvantaging poorer individuals/families (and taxi drivers in particular) who are more likely to have non-compliant vehicles, despite the mitigations put in place within the B-CAP (e.g. grant packages to support upgrade of vehicles and exemptions for those living in the district and those who visit the zone regularly). There are likely to be a range of other unanticipated outcomes which we plan to fully document and record as part of our process evaluation, using information from our semi-structured interviews with stakeholders, documentary analysis of documents related to implementation and focus groups with members of the general public. This period of data collection will be completed by 1 year post implementation and a summary of unanticipated outcomes will be presented at the Bradford Air Quality Programme Board.

## Public engagement

Our Patient and Public Involvement &Engagement (PPIE) approaches have followed the WHO guidance which states: *“cities should demonstrate increased public participation in the decision making processes that affect health in the city, thereby contributing to the empowerment of local people”* (p.6 ) [73]. Initially, one of the authors (SI), presented information about the purpose of the study to a community research advisory group (CRAG). The remit of this group is to consider and advise researchers from the vantage-point of the people who will be most affected by any research or implementation plans –communities themselves. The CRAG considered the topic of tackling air quality as a high priority and encouraged us to formulate plans with community perspectives as a central focus of the research. We sought to achieve this through arranging three art-based community workshops with members of the general public and elected ward councillors. These workshops held two broad objectives: first to ascertain community views about acceptability of and potential impact of the CAZ, and second, to explore what kinds of research questions and general areas of exploration should be included as part of the evaluation.

The outputs from these art-based workshops provided important insights about potential consequences associated with each of the four CAZ options which then generated some useful research ideas. For example, attendees advised us to monitor children’s exposure to air pollution on the route to and from school and to explore what factors people take into consideration when deciding how they will travel to school. We were further informed that raising awareness about air quality was as important as conducting research about it, which prompted us to create a short film^1^ on this subject which has been widely viewed and attracted favourable comments. Equally, we have engaged with our news media (TV, both local and national, radio and print) which has further raised awareness about air quality and this study.

Our PPIE work has evolved from generic engagement with members of the public, as described above, towards the creation of a PPIE group specifically focussed on air quality research and implementation plans. This group is titled Pollution Research Advisory Group (PRAG) and is comprised of 12 members of the public who all live within the boundary of the proposed CAZ area. This group meets regularly to provide advice and guidance to the various work packages as part of the BIB Breathes study.

## Dissemination and impact

We are committed to sharing our learning as widely as possible using a variety of channels. Air quality is one of the most pressing health issues faced by our country, and public interest in this issue is rising. We will develop bespoke dissemination, knowledge exchange and impact plans for our key stakeholder groups. For National (DEFRA; Office for Health Improvement and Disparities; NICE; Local Government; Clean Air Groups; Active Travel groups) and regional (local authorities, schools) stakeholders, we will produce policy and parliamentary briefing notes, plain English summaries and hold dissemination events. We will develop implementation guidance for other local authorities, highlighting successful implementation strategies. For academic audiences, we will publish our findings in high impact open access journals and present at relevant national and international conferences.

We have an exciting opportunity to disseminate our findings creatively to families and children. Building on our citizen science air quality monitoring, we will work closely with teachers to develop school based curriculum materials which will allow pupils to develop air quality monitoring schedules and use these to develop and evaluate their own local initiatives to improve air quality. We will develop a web data dashboard allowing citizen scientists to view their air quality measurements on an interactive map, and compare with other areas. We hope to use these materials as a springboard to engage pupils, particularly from disadvantaged backgrounds, to engage in science, technology, engineering and mathematics (STEM) learning. We will hold engagement events with parents and teachers from participating schools outside of intensive measurement periods to raise awareness of issues related to air quality.

We will use a range of communication channels to inform the general public about our work. At a national level we will engage closely with our media partners (including BBC Radio 4, The Guardian) to promote relevant press coverage of key findings. We will produce a range of blogs and video summaries and disseminate these widely using our established social medial channels (Twitter, Facebook, and YouTube). For local communities, we will hold a range of engagement events in community venues (schools, libraries, mosques, community centres) to give a summary of our findings.

## Discussion

We have a rare opportunity to conduct a quasi-experimental evaluation of a city-wide air quality intervention (Bradford Clean Air Plan: B-CAP), which includes implementation of a charging Clean Air Zone (CAZ) to determine its impact on air quality, health outcomes and inequalities across the life-course. Our proposed study is unique in that it additionally includes a full process and implementation evaluation in addition to planned cost-effectiveness analysis. This will provide rich learning to other areas who are planning to implement this type of approach to improve air quality.

The COVID pandemic has been a major challenge to both implementation of B-CAP and the research programme, causing delays in fieldwork and implementation of the B-CAP. Data collection started in Summer 2021 after lock down periods instigated by the pandemic were lifted, approximately 12 months later than planned. However, the corresponding delay in implementation of the plan has meant we have still been able to collected baseline data. We have had to amend our data collection tools and analysis plans to take into account changes in travel behaviour as a result of the pandemic (for example, reduction in travel during initial lockdown periods resulting in improved air quality for a temporary period, decreased use of public transport as lockdown restrictions eased) as well as periods of reduced health service use affecting measurement of our primary outcome. A strength of our study is its use of routinely collected data available from the cBradford dataset which has remained unaffected by the pandemic, and our use of an interrupted time series design with segmented regression break points which means we are able to isolate ‘atypical’ periods during the pandemic lockdown and account for these in our analyses.

Our research has potential for great impact. The UK Government have committed over £475 million to tackle air quality, primarily through encouraging adoption of charging CAZs across the UK. However, as yet there is no evidence exploring the impact of these types of initiatives on health. Our findings will allow those implementing similar initiatives to model the health impact within their own areas, and our health economic evaluation will enable informed decisions about value for money of such schemes. We also anticipate that our research will have impact for communities, increasing knowledge about the effects of air quality, providing strategies to reduce air pollution exposures, helping to reduce health inequalities, and promoting wellbeing.

## Supplementary Information

**Additional file 1: Supplemental file 1.** Bradford Clean Air Plan Logic and Dark logic model. **Fig. S1.** 1 Logic model for the Bradford Clean Air Zone. **Fig. S1.** 2 Dark logical model for the Bradford Clean Air Plan: Potential adverse events and unintended consequences of the B-CAP.

**Additional file 2: Supplemental file 2.** Baseline travel questionnaire (v5 19.04.2021).

## Data Availability

Data will be available via the Born in Bradford executive group.

## Abbreviations

ANPR: Automatic Number Plate Recognition
AURN: Automatic Urban and Rural Network
B-CAP: Bradford Clean Air Plan
BiB: Born in Bradford
CAZ: Clean Air Zone
CM: Continuous Monitoring
DEFRA: Department for Food, Environment and Rural Affairs
HGV: Heavy Goods Vehicle
ITS: Interrupted Time Series
LGV: Light Goods Vehicle
NO_2_: Nitrogen Dioxide
PM: Particulate Matter
QALY: Quality Adjusted Life Years
SES: Socio-economic Status
SME: Small Medium Enterprises
T: Temperature
WP: Work Package

## Footnotes

1 https://youtu.be/nV-0UEEsMzI
