# Supplementary material for: Study Protocol. Evaluating the life-course health impact of a city-wide system approach to improve air quality in Bradford, UK: A quasi-experimental study with implementation and process evaluation
Source: Environ Health. 2022 Dec 5;21:122. doi: 10.1186/s12940-022-00942-z (PMC9720926; doi:10.1186/s12940-022-00942-z)
Supplement: Supplementary file 1 — Additional file 1: Supplemental file 1. Bradford Clean Air Plan Logic and Dark logic model. Fig. S1. 1 Logic model for the Bradford Clean Air Zone. Fig. S1. 2 Dark logical model for the Bradford Clean Air Plan: Potential adverse events and unintended consequences of the B-CAP. [file 12940_2022_942_MOESM1_ESM.pdf]

Supplemental file 1: Bradford Clean Air Plan Logic and Dark logic model

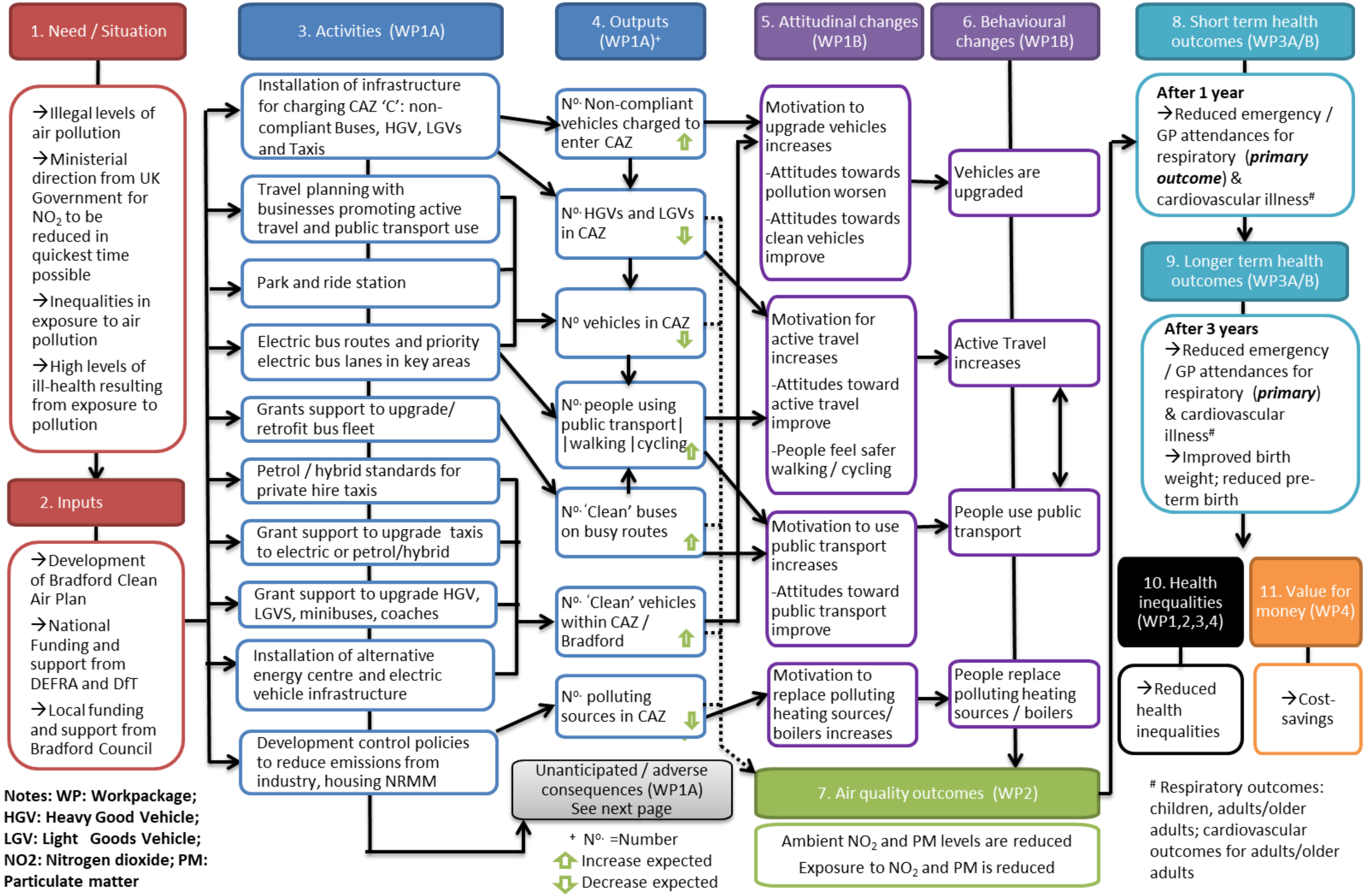

Figure S1.1 Logic model for the Bradford Clean Air Zone

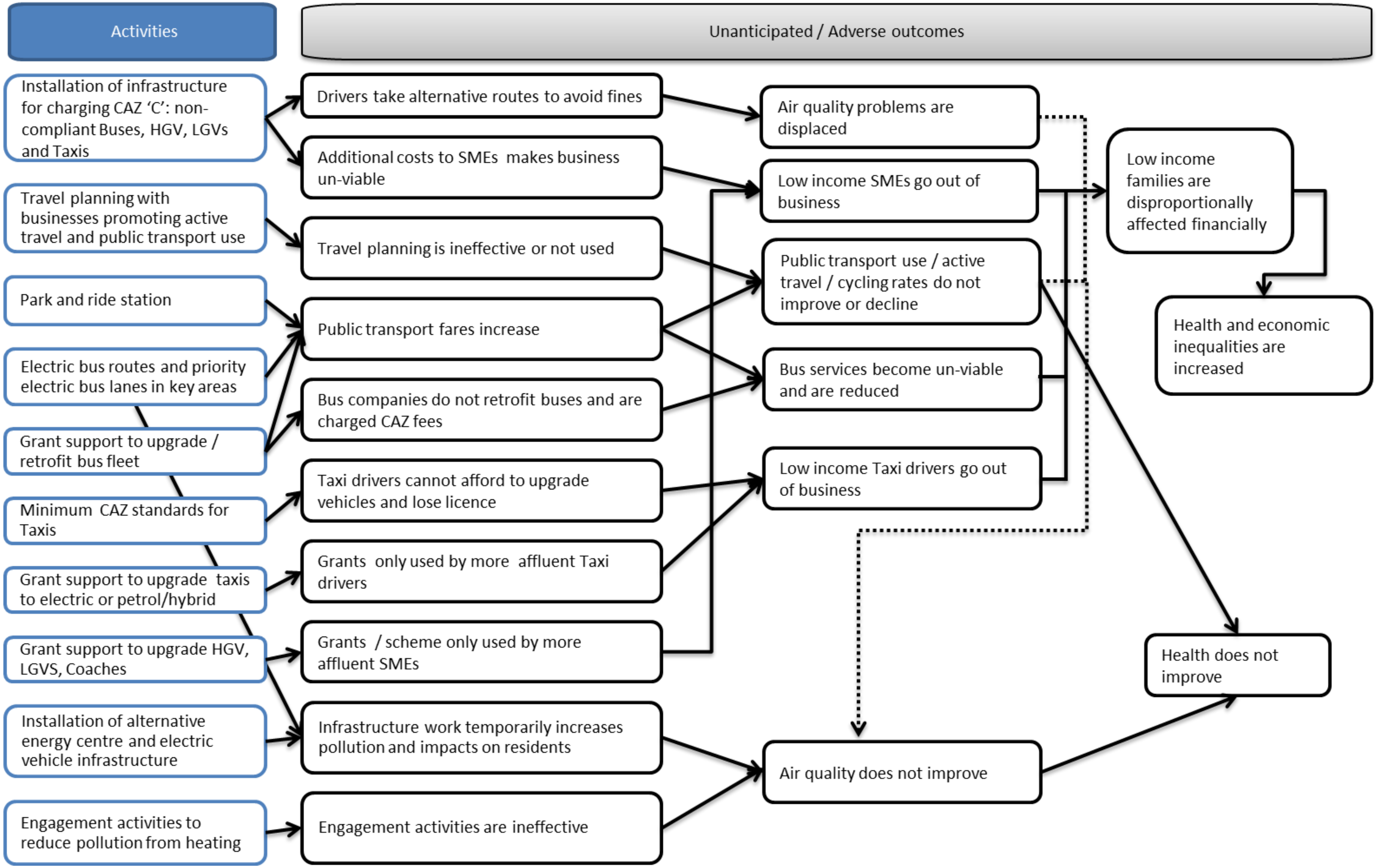

Figure S1.2 Dark logical model for the Bradford Clean Air Plan: Potential adverse events and unintended consequences of the B-CAP
