# Supplementary material for: Study Protocol. Evaluating the life-course health impact of a city-wide system approach to improve air quality in Bradford, UK: A quasi-experimental study with implementation and process evaluation
Source: Environ Health. 2022 Dec 5;21:122. doi: 10.1186/s12940-022-00942-z (PMC9720926; doi:10.1186/s12940-022-00942-z)
Supplement: Supplementary file 2 — Additional file 2: Supplemental file 2. Baseline travel questionnaire (v5 19.04.2021). [file 12940_2022_942_MOESM2_ESM.pdf]

Born In Bradford BiB Breathes Questionnaire

1. **What is your postcode?**

2. **If you do not live in Bradford Metropolitan District, do you travel to Bradford regularly for work, study or leisure?**

Yes	
No	
Not Applicable	

Air Quality

The next section is asking questions around your thoughts on Air Quality.

Air Quality is the term we use to describe how polluted the air we breathe is.

3. **What do you think about the air quality in Bradford generally?**

Very Poor	Poor	Fair	Good	Excellent
1	2	3	4	5

4. **How do you think this compares to other parts of the UK?**

Better	
About the Same	
Worse	
Don't Know	

5. **How concerned are you about air quality in Bradford?**

Not at all Concerned	Slightly Concerned	Somewhat Concerned	Moderately Concerned	Extremely Concerned	Don't know
1	2	3	4	5	6

6. **Do you think other people in Bradford are concerned about air quality?**

Not at all Concerned	Slightly Concerned	Somewhat Concerned	Moderately Concerned	Extremely Concerned	Don't know
1	2	3	4	5	6

7. **How important do you think it is to improve air quality?**

Not at all Important	Slightly Important	Neutral	Moderately Important	Extremely Important	Don't know
1	2	3	4	5	6

8. **Whose responsibility do you think it is to improve air quality in Bradford? (tick all that apply)**

Government	
Council	
General Public	
Factory Owners	
Other Business Owners	
Other (Please Specify)	

9. **How do you think levels of air pollution have changed since the start of the pandemic (e.g. since March 2020)?**

Increased	
Stayed the Same	
Decreased	
Don't Know	

Clean Air Zone (CAZ)

Now we want to ask you about Bradford Councils Plans to reduce pollution in the city, also known as [Breathe Better Bradford](#).

Along with other cities with high levels of pollution, Bradford is planning to implement a 'Clean Air Zone' in the city where older polluting buses, coaches, taxis, heavy goods vehicles and light goods vehicles that enter parts of the city will be charged. Diesel vehicles will need to be Euro 6 emission standard (e.g. built in 2015 or later) and Petrol Euro 4 emission standard (e.g built in 2005 or later) as a minimum to avoid a daily charge. Daily charges will be £50 for heavy goods vehicles, coaches and buses, £12.50 for taxis and £9 for light goods vehicles and minibuses. The council are offering a range of incentives to help taxi and transport operators upgrade to cleaner hybrid or electric vehicles along with other initiatives to improve air quality. Private vehicles (e.g. cars and motorcycles) are exempt from the clean air zone charges.

The map below shows the proposed charging areas for the Breathe Better Bradford Clean Air Zone. This includes all areas of Bradford which currently exceed legal limits of pollution.

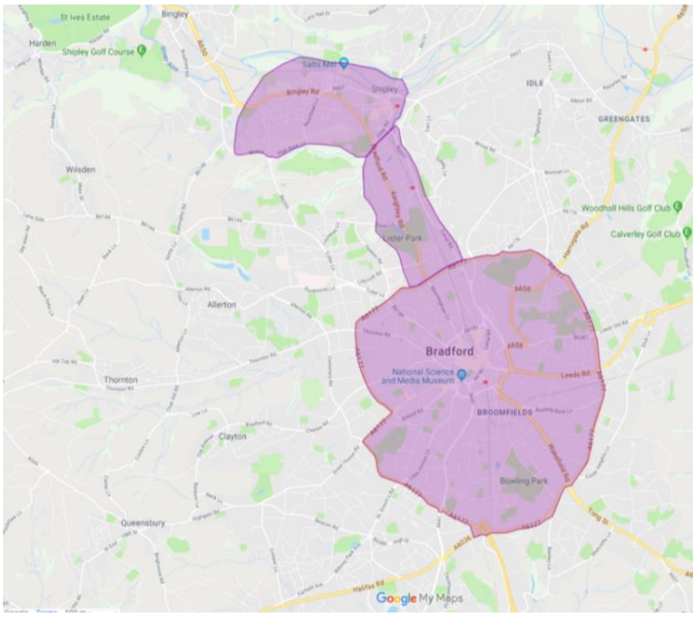

10. **Have you heard about the Clean Air Zone before this survey (also known as Breathe better Bradford)?**

Yes	
No	
Don't Know	

11. Do you think it is a good idea?

Yes	
No	
Don't Know	

12. To what extent do you agree or disagree that the proposed Clean Air Zone covers the right areas of Bradford? (Please Circle)

Strongly Disagree	Disagree	Neither Agree nor Disagree	Agree	Strongly Agree
1	2	3	4	5

13. If you disagree, which areas do you think should be included? (free text)

……………………………………………………………………………………

14. Do you think the Clean Air Zone is going to reduce the air pollution in Bradford?

Yes	
No	
Don't Know	

15. The next few questions are about charging different vehicles to drive in the Clean Air Zone.

Do you think it is a good idea to charge this type of vehicle?	Yes- good idea to charge them	No	Don't Know
Buses and Coaches			
Taxis			
Vans			
HGVs (Heavy Good Vehicles, weighs over 3.5 tonnes)			
Private Cars			

16. Would you be more likely to use a taxi if it was electric compared to a diesel or petrol one?

Yes	
No	
Don’t Know	

17. There are plans to offer grants to some taxi firms and local small to medium size business to help them upgrade their vehicles or switch to electric vehicles. Do you think offering these grants will be helpful for uptake of electric vehicles?

Yes	
No	
Don’t Know	

18. How much do you agree or disagree with the following statements about the Clean Air Zone: (Please Circle)

	Strongly Disagree	Disagree	Neither Agree nor Disagree	Agree	Strongly Agree
It will improve the health of my family and community	1	2	3	4	5
The Council has consulted well with the public about the plans for it	1	2	3	4	5

19. Is there anything else you would like to tell us about your thoughts on the CAZ or air quality more generally? (free text)

……….

Employment and Job Security of the Main Earner

We are interested in what type of work you do. A lot of people's work has been affected by coronavirus. We would like to know how your family has been affected. To make it easier to answer these questions, we are asking about the person who usually contributes the most money to your household. If you and your partner contribute the same amount, please think of yourself when answering these questions.

20. Who is currently the main earner in the household?

Me	
My Partner	
Other Household Member	
Me and my Partner earn the same amount	

21. Is the main earner in your household currently…..

Employed	
Employed but not working (on furlough)	
Employed on the wage subsidy scheme (e.g., working less hours with wage topped by government	
Self-employed and working	
Self-employed and not working	
Unemployed	
Other (please specify)	
Don't know	

22. If the main earner is currently working, are they mainly working from home or going out to work?

Working from home	
Going out to work	

23. Has anyone in your household lost their job since the pandemic started (i.e., since March 2020)? Please tick all that apply.

Me	

My Partner	
Other Household Member	

24. How much do you agree or disagree with the following statements today: (tick one)

	Strongly Disagree	Disagree	Neither Agree nor Disagree	Agree	Strongly Agree
I worry about the job security of the main earner	1	2	3	4	5

25. In the next 12 months do you expect the income of the main earner is likely to be uncertain?

Yes	
No	
Don't know	

26. In 12 months' time, do you expect the main earner to still have their job?

Yes	
No	
Don't know	

27. Which of these best describes the sort of work the main earner usually does?

Senior managers or administrators (usually responsible for planning, organising and co-ordinating work, and for finance) such as: finance manager – chief executive	
Modern professional occupations such as: teacher – nurse - physiotherapist – social worker – welfare officer – artist– musician – police officer (sergeant or above) – software designer	
Middle or junior managers such as: office manager – retail manager – bank manager – restaurant manager – warehouse manager – publican	
Clerical and intermediate occupations such as: secretary – personal assistant – clerical worker – office clerk – call centre - agent – nursing auxiliary – nursery nurse	
Traditional professional occupations such as: accountant - solicitor – medical practitioner – scientist – civil/mechanical engineer Technical and craft occupations such as: motor mechanic - fitter – inspector – plumber – printer –	

tool maker – electrician – gardener – train driver	
Semi-routine manual and service occupations such as: postal worker – machine operative – security guard – caretaker - farm worker – catering assistant – receptionist – sales – assistant	
Routine manual and service occupations such as: HGV driver – van driver – cleaner – porter – packer – sewing machinist – messenger – labourer – waiter/waitress – bar staff	
Other (please write in)	

28. Does anyone in the household specifically have the following profession which involves driving? (tick all that apply)

	Yes- Employed	Yes- Self-Employed	Not Applicable
Private hire or taxi vehicle			
Light goods vehicles driver (e.g. delivery person)			
Heavy goods vehicle driver (including bus/coach/lorry driver)			

Travel

We'd now like to ask you about how you travel around Bradford District and whether this has changed since the beginning of the pandemic.

29. Getting to work

	Petrol/ Diesel Car/Van	Electric/ Hybrid Car	Bus	Walk	Bicycle	Taxi	Train	Other (Please Specify)	Work from Home	Not Applicable
Pre-pandemic (e.g. before March 2020), how did the main earner in your household normally get to work? (Tick all that apply)										
How does the main earner normally get to work now? (Tick all that apply)										

30. Travelling to School:

	Petrol /Diesel Car/Van	Electric/ Hybrid Car	School Bus	Other Bus	Walk	Bicycle	Taxi	Train	Other (please specify)	Not Applicabl e
Pre-pandemic (e.g. before March 2020) how did your children normally travel to school (Tick all that apply)										
How do your children normally travel to school now? (Tick all that apply).										

31. General travel around Bradford District, for example for shopping or leisure activities

	Petrol	Electric/	Bus	Walk	Bicycle	Taxi	Train	Other (please	Not

	/Diesel Car/Van	Hybrid Car						specify)	Applicable
Pre-pandemic (e.g. before March 2020), how did you normally travel around Bradford District for things other than getting to work or school (Tick all that apply)									
How do you normally travel around Bradford now? (Tick all that apply)									

32. If you have changed how you travel due to the Covid-19 pandemic; how likely is it that this change will continue after the lockdowns? (Please Circle)

Not at all likely	Not very likely	Neither likely nor unlikely	Very likely	Extremely likely
1	2	3	4	5

33. Thinking about all your activities in the last week, how much did you and your family household spend on the following transport costs?

Petrol / Diesel / other fuel:

£0	£1-10	£11-20	£21-30	£31-40	More than £40

Taxis:

£0	£1-10	£11-20	£21-30	£31-40	More than £40

Buses:

£0	£1-10	£11-20	£21-30	£31-40	More than £40

Trains:

£0	£1-10	£11-20	£21-30	£31-40	More than £40

34. How often do you use your car for journeys of 0.5 miles or less? 0.5 miles is roughly the distance of a 10 minute walk. (Please Circle)

Never	Rarely	Sometimes	Often	All the Time
1	2	3	4	5

Your Household Essentials

The next questions are about food and money.

35. Please read each statement below and tell us whether the statement was often true, sometimes true, or never true for you or anyone in your household in the last 3 months.

	Often True	Sometimes True	Never True	Don't Know	Do not wish to answer
The food that (I/we) bought just didn't last and (I/we) didn't have money to get more	1	2	3	4	5
(I/we) couldn't afford to eat balanced meals.	1	2	3	4	5

36. Did you or other adults in your household ever cut the size of your meals or skip meals because there wasn't enough for food?

Yes	
No	
Don't know	
Do not wish to answer	

If you answered "YES", how often did you or other adults cut the size of meals or skip meals?

Every week	
Not every week but at least once a month	
Less than once a month but a few times	
Don't know	
Do not wish to answer	

37. Did you or other adults ever eat less than you felt you should because there wasn't enough money for food?

Yes	
No	
Don’t know	
Do not wish to answer	

38. Were you ever hungry but didn’t eat because there wasn’t enough money for food?

Yes	
No	
Don’t know	
Do not wish to answer	

39. Have you or your family received any food from any of the following people/organisations (tick all that apply for each time period.)

	Pre-pandemic (Before Mar 2020)	During the first Lockdown (Mar-Jun 2020)	During the latest lockdown (Jan-Mar 2021)	Now (in the last month)
Food Banks				
Free Food provided by Schools				
Bradford Street Food				
Meals on Wheels				
Food from Faith Based Organisations				
Food from Friends/Neighbour				
Food from Family (not living in your home)				
Other				

40. How well would you say you are managing financially right now?

Living comfortably	
Doing alright	
Just about getting by	
Finding it quite difficult	
Finding it very difficult	
Don't know	
Do not wish to answer	

41. Sometimes people are not able to pay every bill when it is due. Are you currently up to date with all bills?

Yes	
No	
Don't know	
Do not wish to answer	

42. How much do you agree/disagree with the following statements today? Please choose just one option for each statement.

	Strongly Disagree	Disagree	Neither Agree or Disagree	Agree	Strongly Agree
I worry about paying the rent/mortgage	1	2	3	4	5
I worry about getting evicted/having my house repossessed.	1	2	3	4	5

Wellbeing

Next we would like to ask some questions about your mental health and wellbeing.

43. Overall, to what extent do you feel that the things you do in your life are worthwhile? (Where 0 is 'not at all worthwhile' and 10 is 'completely worthwhile).

Not at all Worthwhile										Completely Worthwhile
0	1	2	3	4	5	6	7	8	9	10

44. Over the last 2 weeks, how often have you been bothered by any of the following problems?

	Not At All	Several Days	More Than Half of the Days	Nearly Every Day
Little interest or pleasure in doing things				
Feeling down, depressed, or hopeless				
Trouble falling or staying asleep, or sleeping too much				
Feeling tired or having little energy				
Poor appetite or overeating				
Feeling bad about yourself – or that you are a failure or have let yourself or your family down				
Trouble concentrating on things, such as reading the newspaper or watching television				
Moving or speaking so slowly that other people could have noticed? Or the opposite – being so fidgety or restless that you have been moving around a lot more than usual				

45. If you checked off any problems, how difficult have these problems made it for you to do your work, take care of things at home, or get along with other people?

Not at all	
Several Days	
More than half the days	

Nearly every day	

46. Over the last 2 weeks, how often have you been bothered by any of the following problems?

	Not At All	Several Days	More Than Half the Days	Nearly Every Day
Feeling nervous, anxious, or on edge				
Not being able to stop or control worrying?				
Worrying too much about different things?				
Trouble relaxing?				
Being so restless that it is hard to sit still?				
Becoming easily annoyed or irritable?				
Feeling afraid as if something awful might happen?				

47 Under each heading, please tick the ONE box that best describes your health **TODAY**.

MOBILITY
- I have no problems in walking about ❑
- I have slight problems in walking about ❑
- I have moderate problems in walking about ❑
- I have severe problems in walking about ❑
- I am unable to walk about ❑

SELF-CARE
- I have no problems washing or dressing myself ❑
- I have slight problems washing or dressing myself ❑
- I have moderate problems washing or dressing myself ❑
- I have severe problems washing or dressing myself ❑
- I am unable to wash or dress myself ❑

USUAL ACTIVITIES *(e.g. work, study, housework, family or leisure activities)*
- I have no problems doing my usual activities ❑
- I have slight problems doing my usual activities ❑
- I have moderate problems doing my usual activities ❑
- I have severe problems doing my usual activities ❑
- I am unable to do my usual activities ❑

PAIN / DISCOMFORT
- I have no pain or discomfort ❑
- I have slight pain or discomfort ❑
- I have moderate pain or discomfort ❑
- I have severe pain or discomfort ❑
- I have extreme pain or discomfort ❑

ANXIETY / DEPRESSION
- I am not anxious or depressed ❑
- I am slightly anxious or depressed ❑
- I am moderately anxious or depressed ❑
- I am severely anxious or depressed ❑
- I am extremely anxious or depressed ❑

© 1990 EuroQol Group. EQ-5D™ is a trade mark of the EuroQol Group

Your Worries and Concerns

The last section of the questionnaire is for you to tell us about your recent worries, concerns and positive

experiences.

48) What are your three biggest worries right now? (Please write your biggest worry first).

Worry 1 :____

Worry 2: ____

Worry 3: ____

49) Can you tell us about a challenge you have faced in the last two weeks?

____

50) Can you tell us whether there are any parts of your life that have continued to be easier or more

enjoyable since lockdown first began in March? ____

ADDITIONAL QUESTIONS FOR NON BiB PARTICIPANTS

X1. What is your age?

___ years ☐ Prefer not to say

X2. Are you

☐ Male ☐ Female

☐ I prefer to use my own term ☐ I prefer not to say

X3. What is your ethnic group? (please choose only one of the following, you can be more specific in the next part of this question)

☐ White ☐ Asian/Asian British

☐ Black/ African/Caribbean/Black British ☐ Mixed/Multiple ethnic groups

☐ Arab/Middle Eastern ☐ I prefer not to say

☐ I don't know my ethnicity ☐ If none of these categories are applicable, how would you describe your ethnic origin?

Please state: ____

What do you consider your cultural background? (Only answer this question if you answered 'White' at question X3)

☐ English/Welsh/Scottish/Northern Irish/British

☐ Irish

☐ Gypsy or Irish Traveller

☐ White; Polish

☐ White; Czech

☐ White; Romanian

☐ White; Slovakian

☐ White; Roma

☐ Any other White background (please write)

____

What do you consider your cultural background? (Only answer this question if you answered 'Black/ African/Caribbean/Black British' at question X3)

☐ African

☐ Caribbean

☐ Any other Black background (please write)

____

What do you consider your cultural background? (Only answer this question if you answered

'Asian/Asian British' at question X3)

☐ Indian

☐Pakistani

☐Bangladeshi

☐ Chinese

☐ Any other Black background (please write)

____

What do you consider your cultural background? (Only answer this question if you answered 'Mixed/Multiple ethnic groups' at question X3)

☐ White and Black Caribbean

☐ White and Black African

☐ White and Asian

What do you consider your cultural background? (Only answer this question if you answered 'Other ethnic group at question X3)

☐ Arab/Middle Eastern

If none of these categories are applicable, how would you describe your ethnic origin? (please state)

X4. We would like to contact you again in about 2 years time to ask about your views on air quality again at that point. If you are happy to do this please complete the details you are happy for us to contact you by below . We will store your personal details securely and will not pass them on to anyone else. You can read more about how we keep your information safe here: https://borninbradford.nhs.uk/what-we-do/how-we-use-your-information/

Name:

Address:
Phone:
Email address:

*****END OF QUESTIONNAIRE****
